# Evidence of application of the Basic Antenatal Care principles of good care and guidelines in pregnant women’s antenatal care records

**DOI:** 10.4102/phcfm.v8i2.1016

**Published:** 2016-05-31

**Authors:** Ngxongo T.S. Patience, Maureen N. Sibiya, Nomthandazo S. Gwele

**Affiliations:** 1 Department of Nursing, Faculty of Health Sciences, Durban University of Technology, South Africa

## Abstract

**Background:**

Basic Antenatal Care (BANC) is an approach that is used in the public health institutions of South Africa to provide health care services to pregnant women. The approach was introduced as a quality improvement strategy based on the belief that good quality Antenatal Care (ANC) could reduce maternal and perinatal mortalities and improve maternal health.

**Aim:**

The aim of this study was to analyse pregnant women’s ANC records for evidence suggesting that the BANC principles of good care and guidelines were being applied.

**Setting:**

The study was conducted in the 12 primary health care clinics that were providing ANC services using the BANC approach in eThekwini district, KwaZulu-Natal.

**Methods:**

A cross-sectional quantitative design was used to conduct the study. Data were collected through retrospective record review of 1200 maternity case records of the pregnant women who had attended for ANC services three or more times and was analysed using Statistical Package for Social Sciences (SPSS) version 21.0.

**Results:**

The majority of the processes detailed in the guidelines and principles of good care were evident in the records. However, several were missing or recorded in few reviews. These included the ANC plan, delivery plan, midwives’ counter signatures on the cards, assessment for foetal congenital abnormalities, and consent for HIV testing.

**Conclusion:**

The study identified evidence of incomplete application of the BANC principles of good care and guidelines in pregnant women’s antenatal care records which indicated that the BANC approach was not being successfully implemented. Recommendations were made with regards to policy development, institutional management and practise, nursing education, and further research to assist in successful implementation of the BANC approach in line with the guidelines and principles of good care.

## Introduction

Basic Antenatal Care (BANC) is an approach that is used in the public health institutions of South Africa to provide health care services to pregnant women according to the National Department of Health (NDoH). The approach is listed as one of the priority interventions for reducing maternal and child mortality in this country.^1^ The BANC approach was introduced as a quality improvement strategy based on the belief that good quality Antenatal Care (ANC) could reduce maternal and perinatal mortalities and improve maternal health aiming to achieve Millennium Development Goals 4 and 5.^2^

Until 2007 South Africa used the traditional approach to ANC. The traditional ANC service model was developed in the early 1900s. This model assumed that frequent ANC visits, and classifying pregnant women into low- and high-risk groups by predicting potential obstetric complications, was the best way to care for the mother and the foetus.^2^ The use of the traditional ANC approach in South Africa was prescribed by the South African Nursing Council in the scope of practise for midwives (Regulation R2598 of 1987 as amended by Regulation R260 of 1991).^3^

South Africa adopted the BANC approach from the Focussed Antenatal Care (FANC) model.^2^ The FANC approach is a goal-oriented ANC approach that was recommended by researchers during 2001 and adopted by the WHO in 2002.^4^ The WHO designed and tested a FANC package that included only counselling, examinations, and tests serving an immediate purpose and having a proven health benefit as an ideal approach to be used by developing countries.^5^ South Africa modified the FANC approach to suit the South African circumstances.^6,7^ This followed the realisation by the NDoH that the traditional ANC approach was not working well for South Africa. In 2007, the NDoH advised that all health facilities providing ANC services had to adopt the BANC approach by the end of 2008.^8^

The NDoH provided training for the lead trainers from all the provinces and made available various documents such as handbooks, guidelines, and guides for facility managers.^9,10^ The lead trainers were expected to cascade the training into their respective provinces and to institute and facilitate the implementation of the BANC approach.

Although the BANC approach is seen as a positive measure to improve the quality of ANC in primary health care (PHC) clinics,^11^ a constant rise in maternal and perinatal mortality rates is still the case in South Africa.^12^ Improvement in access to good quality ANC services could make a major contribution towards reducing perinatal and child deaths.^13^ The majority of the avoidable causes of maternal deaths could be addressed by the implementation of the BANC approach.^14^ These include a number of health provider-related issues such as poor initial assessments, problems with recognising problems, delays in referring the pregnant women to different health care facilities causing pregnant women to be managed at inappropriate health care levels, incorrect management, substandard management/care, and failure to take actions when abnormalities were found.^14^ Therefore, establishing the evidence in the clients’ records suggesting that BANC principles and guidelines are being applied would give an indication of how the BANC approach was being implemented and could identify strengths and limitations that would be useful in developing the strategies to facilitate the implementation of BANC services.

### Research aim

The aim of this study was to analyse pregnant women’s ANC records for evidence suggesting that the BANC principles of good care and guidelines were being applied.

### Research questions

The question to be answered was: Is there evidence in the clients’ records suggesting that BANC principles and guidelines are being applied?

## Method

### Study design

A cross-sectional quantitative design was used to conduct the study.

### Setting

The study was conducted in the eThekwini district (formerly known as Durban), one of the 11 health districts of the KwaZulu-Natal (KZN) province in South Africa. The settings for recruitment were the clinics that were providing ANC services using the BANC approach. In total 96 of the 103 PHC clinics in eThekwini district were providing ANC services and were using the BANC approach.^15^

### Study population and sampling strategy

In total (12.5%, *n* = 12) PHC clinics were randomly selected from the 96 PHC clinics that were providing ANC services and implementing the BANC by means of selecting every eighth PHC clinic on the list. This allowed for every one of the 96 PHC clinics that were providing ANC services and implementing the BANC approach to have an equal chance of being included in the study:^16^

- Inclusion criteria: Only the PHC clinics that were providing ANC services and were implementing the BANC approach.
- Exclusion criteria: All PHC clinics that were providing ANC services but not implementing the BANC approach.
- All PHC clinics that were not providing ANC services.
- All PHC clinics that were used for the pilot study.

### Sampling of maternity case records for retrospective review

In total 1200 maternity case records for the pregnant women who received ANC in the sampled PHC clinics during the time of data collection for the current study (October 2013 to March 2014) were purposively selected. This included selecting 100 records from each of the 12 PHC clinics:

- Inclusion criteria for maternity records: The records of pregnant women who had three or more recorded routine ANC visits.
- Exclusion criteria for maternity records: Records of pregnant women who had fewer than three recorded routine ANC visits.

### Pretesting the research instruments

A pretest was conducted before the commencement of the main study in order to establish reliability and validity of data collection instruments and research procedures.

### Data collection

A checklist adapted from ‘The quality check for Antenatal Record’ was used for the record review.^17^ Reliability and validity were ensured first by inviting midwifery experts to render inputs into the data collection instrument (the checklist), an input of the statistician to determine whether the construct validity was appropriate for statistical purposes, and by conducting a pretest of data from the clinics that were implementing the BANC approach.

### Data analysis

The data from the record reviews were captured and subsequently analysed using the version 21.0 of the Statistical Package for Social Sciences (SPSS) computer program. Four of the six steps of quantitative data analysis which included 1) preparation of data for analysis, 2) description of the sample, 3) testing of reliability of measurement, and 4) exploratory analysis of the data were used.^18^ Frequencies are represented in graphs.

## Ethical considerations

Ethical approval was obtained from the university’s Institutional Research Ethics Committee and permission to conduct the study was granted by the provincial and district offices of the KZN Department of Health and the eThekwini municipality. The researcher ensured that beneficence, respect for human dignity, and justice as the main ethical principles^18^ on which the standards of ethical conduct in research are based were adhered to.

## Results

### Recording system used

Of the 1200 maternity case records reviewed (98.4%, *n* = 1181) were the standard white maternity case records cards and (1.6%, *n* = 19) were not; they were the old green maternity case record cards that had been used previously. The colour of the record was not the issue but the structure and information contained in the record. Calling them by colour (white or green) has been a trend in the health services which has been used for identification purposes.

### Antenatal care consultation processes

Reviews of ANC records were conducted to check whether selected processes were implemented during ANC consultations. Some processes are general and apply to all ANC visits, whereas others are recorded initially during the first visit and are subsequently adjusted as the needs arises during follow-up visits.

### General antenatal care consultation processes

**FIGURE 1:**
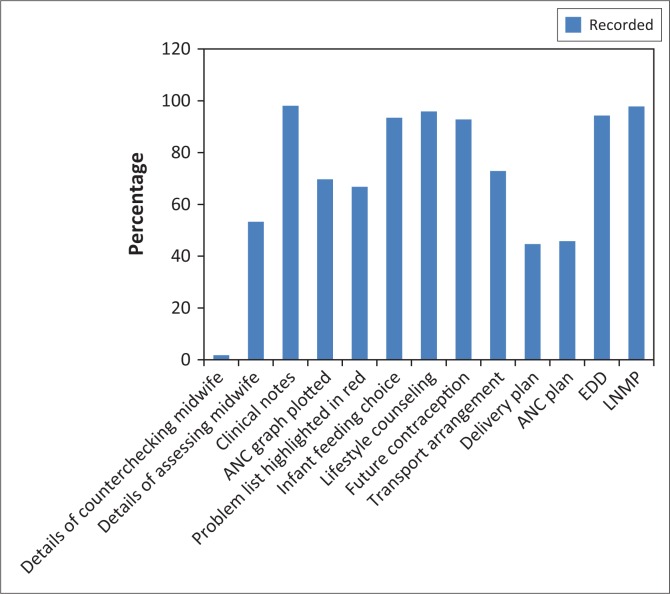
General ANC consultation processes.

The assessment with regards to the general ANC consultation processes revealed that there were more reviews with last normal menstrual period (LNMP), expected date of delivery (EDD), transport arrangements, future contraception, lifestyle counselling, infant feeding choice, ANC graphs, clinical notes, and signature of the midwife who assessed the pregnant women (54% – 98%, *n* = 639–1177) recorded than those without such records. LNMP was recorded in the highest number of reviews (98%, *n* = 1177). The ANC plan, delivery plan, and midwives’ counter signatures on the cards were recorded in fewer reviews compared to other elements (2% – 46%, *n* = 22–550) with the midwife countersigning the card being the least frequently recorded element (2%, *n* = 22). Figure 1 presents the findings in percentages of how the information was recorded.

### Assessment for conditions

According to the BANC principles of good care and guidelines^6^ every pregnant woman attending ANC should be assessed for the following conditions during every ANC visit as part of the ANC consultation process:

- pre-eclampsia by monitoring of the blood pressure and doing dipstick urinalysis
- HIV infection by doing an HIV test
- malnutrition and anaemia by measuring mid upper arm circumference and monitoring of blood haemoglobin level
- congenital abnormalities by screening for genetic defects
- foetal growth, post maturity, by plotting and interpreting the antenatal graph
- foetal movements by monitoring foetal kick counts.

**FIGURE 2:**
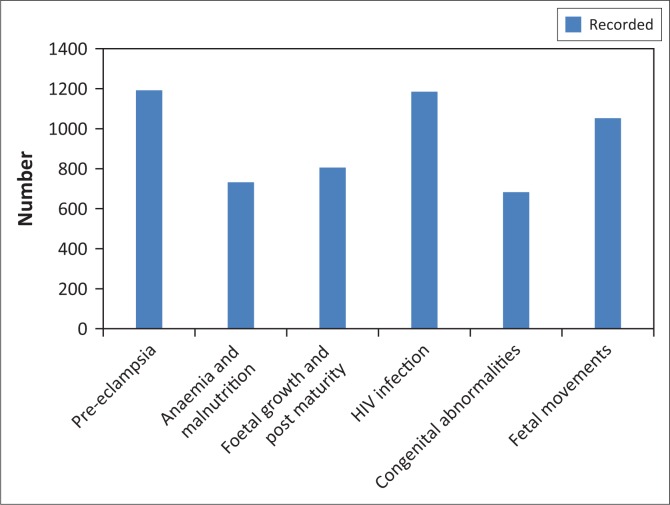
Assessments for conditions in primary health care clinics (*n* = 1200).

All pregnant women should be issued with prophylactic treatment such as haematinics (ferrous sulphate and folic acid) and calcium supplements. The ANC records were reviewed for evidence of the assessment for these conditions and supplying of calcium (Figure 2). These assessments were recorded in (60% – 99%, *n* = 683–1192) of the reviews. Assessment for pre-eclampsia was recorded in the majority of the reviews (99%, *n* = 1192) and assessment for congenital abnormalities recorded in the fewest of the reviews (60%, *n* = 683).

### First visit consultation processes

The assessment with regards to the first ANC visit’s consultation processes included checking whether various activities were conducted, that the dates for tests were set, the results of tests were recorded, and whether actions were implemented based on abnormal findings.

History, routine investigations, HIV screening, physical examination, and supply of calcium supplements to the pregnant women were recorded in the majority of reviews (86% – 100%, *n* = 1031–1200). The other activities and/or tests were recorded in fewer reviews (27% – 70%, *n* = 311–829). The least recorded was consent to HIV testing which was signed in (27%, *n* = 311) of the records only.

The dates when all the activities were performed was recorded in (99.4% – 100.0%, *n* = 459–1200) of the reviews for all activities in all PHC clinics.

The results for the tests and activities were recorded in (97.0% – 99.9%, *n* = 473–1199) of the reviews except for the results of pap smears which were only recorded in (43%, *n* = 197) out of 459 records where pap smears were conducted.

Actions in response to abnormal findings were recorded in more than (80% – 95%, *n* = 156–310) of the reviews for all activities and tests except for TB for which actions were only recorded in (45%, *n* = 128) of the reviews out of 286 reviews that had abnormal findings on TB recorded.

### Follow-up visit consultation

Analysis of the follow-up visits included checking on the timing of repeat tests and assessing whether follow-up actions had been implemented in response to abnormal findings. Both of these processes were not done well because there were no such records in more than 20% of the reviewed documents. All repeat tests that were due during the ANC period such as RPR and HIV were accomplished in due time in (73%, *n* = 878) of the reviews and not accomplished in due time in (27%, *n* = 322) of the reviews. Follow-up actions, in relation to previous findings, were implemented in (59%, *n* = 582) of the records and not implemented in (41%, *n* = 412) of the records. The tests and activities that needed to be repeated were reportedly implemented in due time in (75%, *n* = 898) of the records for routine investigations; (74%, *n* = 519) of the records for HIV screening; and (89%, *n* = 918) of the records for supplying calcium supplements.

## Discussion

### Key findings

Some elements of the guidelines and principles of good care were evident in the records while others were not. Elements such as LNMP, EDD, future contraception, lifestyle counselling, infant feeding choice, transport arrangement, and clinical notes were all recorded well in most of the reviews. The most poorly recorded activities included the ANC and delivery plans and the details of the midwife countersigning the records. Almost all activities and tests conducted during the ANC consultation process were recorded in more than 50% of the reviews except for the pap smear and informed consent to HIV. Assessments for various conditions were recorded in most reviews although assessments for foetal movements, congenital abnormalities, HIV infection in and foetal growth and post maturity were recorded in the least reviews. The majority of activities conducted during the first ANC visit consultation were recorded in less than 50% of the reviews. The most poorly recorded activity was the recording of an assessment whether the pregnant woman was eligible for the BANC approach. The best recorded were all tests and procedures that were due in the first visit consultation. With regards to the follow-up visit, all repeat tests were recorded to have been conducted in due time in most reviews.

### Discussion of the key findings

This record review approach to analyse pregnant women’s ANC records for evidence suggesting that the BANC principles of good care and guidelines were being applied was based on the understanding that, in the nursing profession, what is not recorded is considered not done, that good record keeping is an integral part of nursing and midwifery practise, and is essential for the provision of safe and effective care.^19^

Health policymakers have ensured that South Africa’s goals are clear and that many policies are in place. What is now needed is to track and manage progress in the implementation of these policies.^20^ The guidelines should be made available in all institutions and the staff members should be made aware of and be familiar with them in order for the staff members to implement them.^21^ It is not enough just to have the protocols and guidelines available in the institutions; but midwives and doctors should be trained in the use of these protocols and guidelines.^21^ In the current study some elements of the guidelines and principles of good care were evident in the records while others were not. The question is why: is it because the staff members were not aware of these; were they not trained in implementing the guidelines, or were they intentionally ignoring the guidelines?

A similar situation where the guidelines were not implemented was noted^22^ in Zimbabwe in a study of nurses and midwives which investigated the dilemmas and paradoxes in providing and changing ANC. In this study participants implied that they designed their own ways of coping with situations. One strategy used by nurses and midwives to ease pressure in Zimbabwe was to ignore the government directives and if they were accused of doing things incorrectly, they would work as usual and pretend they had not seen the new regulations. It is important to acknowledge that sometimes policy practises, resource constraints, and community dynamics intersect, creating a complex work situation resulting in a stressful work environment and paradoxes and dilemmas for the caregivers in implementing recommended changes.^22^ When policies are formulated with little reflection on the local conditions within which they are to be implemented, issues of policy and practise and organisational context, within which the staff members care and implemented the proposed changes, could potentially highlight conflicts. It is also critical to keep in mind that implementing change might be difficult if it is not based on realities, knowledge, experiences, and perspectives of those implementing the change and those expected to benefit from it.^22^

In the Zimbabwean study, observations and informal conversations showed that the FANC guidelines did not play a large role in guiding the daily work of health care workers. The health care workers did not know whether the FANC guidelines were actually available at the health facility or not. The ANC card provided an important working tool for the health care workers because it structured the delivery of the ANC services but the ANC card only covered a subset of the services recommended in the FANC guidelines. This could explain why some of the recommended services were not delivered to the women.^22^ The findings highlight the importance of ensuring that the recording card used is designed in line with the guidelines and updated whenever there are changes made in the operating guidelines. This raises a question with regards to the findings of the current study which is whether the ANC record was designed in line with the principles of good care and guidelines. The current study did not address this issue.

In the current study there were certain elements that were recorded more poorly than others. The most poorly recorded activities included the ANC and delivery plans and the details of the midwife countersigning the card which were only evident in (1.8% – 45.8%, *n* = 22–550) of the records. The current study did not investigate the reasons why these three elements were so poorly recorded compared to the others.

The worst recorded aspect was the midwife’s countersignature on the ANC record card. Counterchecking is a wise precaution in most situations and is important to prevent staff members from making errors.^23^ The BANC approach recommends counterchecking the first ANC visit and again during the 32nd week visit. However,^9^ counter checking requires time and it may add up to 20 min to each activity to carry out an independent double check.^24^

This practise is also advocated by the Institute for Safe Medication Practises in their programme called Independent Double Checks.^24^ The selective and proper use of independent double checks can play an important role in practise safety. It is also essential that the staff members be educated about the importance of counterchecking each other and how to carry it out properly as an independent cognitive task and not as a superficial routine task.^24^ A standardised process and tools could be used in order to reduce process inconsistencies and to establish a standard process for carrying out an independent double check.^24^ Double checks should not be used as a means of fixing problems when more fundamental system redesign is needed^24^ and sole reliance on double checks should be avoided because they will sometimes fail for a variety of reasons. There are various other strategies that could be used to ensure that clinics staff members assess their clients correctly and completely.

Another finding indicating lack of evidence of implementing the BANC principles of good care and guidelines was the absence of the record of ANC and delivery plans. The ANC and delivery plans should be prepared in consultation with the women during the first visit and reviewed and updated at each follow-up visit.^9^ Similar recommendations are given by WHO^25^ in the FANC model. It is important that the plans be prepared because the plan defines goals for future direction and determines resources required to achieve those goals.^26^ Having a plan of pregnancy management facilitates management by determining the objectives and highlighting the purposes for which various activities are to be undertaken. This provides a blueprint for a course of action which brings order and rationality. The plan enables the clinic staff members to focus their attention on the objectives or goals when managing the client and, if it is executed properly, it should lead to improved focus, coordinated action, control, and time management.^26^ Planning typically offers a unique opportunity for information-rich and productively-focussed discussions between the health care workers and the clients which provide an agreed context for subsequent management activities.

### Recommendations

It is recommended that relevant policies, service delivery guidelines, and protocols should be available in all PHC clinics that provide ANC services and all staff members and managers should be trained to use these documents to ensure safe and standardised practise. Records should be designed so that relevant aspects of the implementation of policies required are recorded – this will facilitate auditing of the implementation of policies.

Each PHC clinic should have a performance and quality audit team which conducts regular audits and SWOT analyses of how ANC services are being provided. Feedback should be provided to the manager and all the clinic staff members who will thereafter compile implementation plans and action audit reports.

All midwives involved in ANC should have received some training in BANC. Ongoing in-service education for the practising midwives should be available to keep them abreast with the BANC principles of good care and guidelines and the new developments in the BANC approach. It is recommended that a broader study involving other districts and other provinces should be conducted.

It is recommended that a qualitative study using either the interviews or questionnaires be conducted with the nurses involved in ANC services to explore the reasons for some guidelines being poorly implemented.

### Limitations of the study

Data collection did not include gathering information directly from clinic staff members involved in the implementation of the BANC approach. This source of information could have enriched the study’s findings. The results cannot be generalised to the rest of South Africa because of the sample size which was representative of only the eThekwini district, thus the need for a broader study involving other districts and or provinces.

## Conclusion

The review of records showed that there was incomplete implementation of the BANC principles of good care and guidelines which indicated that the BANC approach was not being successfully implemented across the board. This highlighted the need to identify strategies to ensure successful implementation of the BANC approach in order to ensure good quality ANC that can reduce maternal and perinatal mortalities and improve maternal health.
